# Increased lactic acid content associated with extracellular matrix depletion in a porcine disc degeneration induced by superficial annular lesion

**DOI:** 10.1186/s12891-019-2937-x

**Published:** 2019-11-20

**Authors:** Jinhui Shi, Xichao Zhou, Zhen Wang, Swamy Kurra, Junjie Niu, Huilin Yang

**Affiliations:** 1 Department of Orthopedics, The First Affiliated Hospital of Soochow University, 188 Shizi Street, Suzhou, 215006 Jiangsu China; 2 Department of Orthopedics, Suzhou Hospital of Traditional Chinese Medicine, 889 Wuzhongxi Road, Suzhou, 215000 Jiangsu China; 3 Department of Orthopedic Surgery, SUNY Upstate Medical University, 720 E. Adams St, Syracuse, NY 13210 USA

**Keywords:** Lactic acid, Aggrecan, Intervertebral disc degeneration, Nucleus pulposus, Annulus fibrosus

## Abstract

**Background:**

Degenerative intervertebral disc (IVD) disease can cause lower back pain. However, the change of lactic acid content during disc degeneration process still unclear. The objective of this study was to investigate whether the change of the content of lactic acid is associated with depletion of degenerative intervertebral disc extracellular matrix.

**Methods:**

A total of 18 miniature pigs were equally divided into annular lesion surgery (AL) and sham group. The lateral superficial annulus fibrosus (AF) of T12-L4 discs in AL group were penetrated by 3.5 mm trepan with the depth of 3 mm, the same IVD were only exposed without any injury in the sham group. At 4, 8 and 12 weeks after surgery, the degree of intervertebral disc degeneration was evaluated by magnetic resonance, histological and biochemical analysis.

**Results:**

No obvious degeneration was found in sham group. However, disc degeneration was found and gradually worsened in AL group after surgery. Histological analysis showed that the AF was rupture and disorder, the number of cells in nucleus pulposus (NP) was decreased in AL group. Compared with the sham group, the extent of type II collagen (Col-II) and aggrecan in NP tissue was dramatically decreased in AL group, consistent with the results of Col -II immunohistochemistry staining and quantitative reverse transcription polymerase chain reaction (qRT-PCR). Besides, the gene expression of matrix metallopeptidase 3 and 13 also continuous increased in AL group. The amount of lactic acid and nerve growth factor in NP tissue was gradually increased after operation in AL group.

**Conclusions:**

The content of lactic acid gradually increased after annular lesion, associated with the damage of AF structural and the decrease of Col -II and aggrecan in NP tissue, which leading to the disc degeneration. Depletion of extracellular matrix is consistent with lactic acid accumulation inside of IVD.

## Introduction

Chronic low back pain (LBP) is one of the most common causes which affect the quality of life and work ability for many people. It is also the second most common cause of job-related disability in the United States for adults under 45 years of age, and is ranked third in diseases requiring surgery in the United States [1]. Crock et al. first proposed that the degeneration of the internal structure of the intervertebral disc (IVD) is one of the most common mechanical causes of LBP [2].

Degenerative changes in the histology and biochemical properties of IVD includes a loss of proteoglycan and water content in nucleus pulposus (NP), conversion of collagen types, calcification of endplate, increased degradative enzymes, and upregulation of proinflammatory cytokines [3, 4]. Many studies indicated that degenerative disc had an obvious acid microenvironment compare with normal disc. Kitano et al. [5] demonstrated the pH value of central-disc in asymptomatic lumbar discs was 7.14 ± 0.04, while in symptomatic disc disease was 6.65 ± 0.07. Diamant et al. [6] found the intradiscal pH in lumbar degenerative disc varied between 5.7 and 7.5. Wuertz et al. [7] concluded that microenvironmental conditions of pH may be the major limitation for mesenchymal stem cells-based IVD repair. There were several mechanisms of conformation of acid condition in IVD, lactic acid accumulation is one of the main reasons for the decline in pH value [8, 9]. Leakage of the acid from annular fissures to surrounding nerves causes excruciating pain, even scarring nerve roots to cause chemical radiculitis [6, 10]. Eliminating the amount of lactic acid in the intervertebral disc to improve the pH may have potential benefits for degenerative discs. Besides, increased nerve ingrowth is found in the NP during intervertebral disc degeneration (IDD), and has been suggested as a potential contributor to LBP [11–13]. In vitro and in vivo studies found nerve growth factor (NGF) increased during disc degeneration, where it is thought to contribute to both innervation of degenerating discs and neuronal sensitization [12–15].

Although the role of pH in the IDD has gradually become known in recent years [16], however, to our knowledge, no study evaluated the change of lactic acid content during disc degeneration process. The aim of this study is to identify the changes of the lactic acid as well as extracellular matrix of IVD and their roles in IDD by using a porcine model which was induced by superficial annular injury [17].

## Methods

### Experimental animals and surgical procedure

Eighteen female miniature pigs (12 months of age, weight 40 kg) provided by the Laboratory Animal Center of Soochow University, China were used. The animals were housed in units that met the recommended weight-space specification and were provided with water and nutritionally balanced feed. The protocol was approved by the Committee on the Ethics of Animal Experiments of Soochow University (Permission Number: 2017–059).

Animals were randomly divided into sham and annular lesion surgery (AL) groups. Each group had 9 pigs, all the animals were sedated by an intramuscular injection of ketamine (20 mg/kg body weight) and tranquilized (10 mg/kg body weight), and next they were anaesthetized by intravenous injections of 3% pentobarbital sodium (1 ml/kg body weight). The IVD of T12-L1, L1–2, L2–3 and L3–4 were exposed through a left retroperitoneal approach. In the AL group, a 3.5 mm diameter trephine was used to create a lesion on the lateral AF of those four discs. The depth of the lesion was controlled at 3 mm. The fragment of annular fibrous from the lesion was removed, the inner annular fibrous was kept intact and no NP outflow. In the sham group, the lumbar discs were exposed only, without damage to the disc structure. Postoperatively, the animals recuperated in a facility for a period of 12 weeks, where they were monitored daily. At 4, 8 and 12 weeks postoperatively, three animals were randomly selected from the AL and sham groups. For euthanasia, pigs were anaesthetized by intravenous injection of 3% pentobarbital sodium (1 ml/kg body weight), and euthanized by injection of potassium chloride (100 mg/kg). All animals were sacrificed after completing MRI examination, and the T12-L4 section spine specimens were harvested. Each IVD and its upper and lower cartilaginous endplates were retained as a sample. From each animal four samples were collected: T12-L1, L1–2, L2–3 and L3–4 segments. The T12-L1 and L1–2 segments were used in molecular biology experiments; and, the L2–3 and L3–4 were fixed in 10% formalin solution for further histological experiments.

### Magnetic resonance imaging (MRI)

At 4, 8 and 12 weeks after surgery, all animals were scanned using the GE Signa HDxt 3.0 T superconducting MRI system (Signa, General Electric Medical Systems, USA), with a magnetic field gradient of 40 mT/m. The magnetic field switching rate is 150 mT/ms. Sagittal and axial T2-weighted images were obtained. According to Pfirrmann disc degeneration grade [18], discs were assigned 1 of 5 grades. Two MRI diagnostic physicians, who were blinded to this study, assessed the L1-S1 IVD images independently. The assessment was repeated at intervals of 3 weeks.

### Histologic assessment

Each sample was decalcified routinely [19] and then embedded in paraffin and cut in 5 μm slices in the sagittal plane from the central part of the disc which included the mid NP, surrounding annulus and the endplates. Hematoxylin and eosin (HE) staining was used to evaluate the morphological characteristics of the cells in the samples. Masson trichrome staining was used to evaluate the connective tissue and the extracellular matrix of the cartilage. Safranin O and fast green staining was used to evaluate the cartilage and bone tissue. The expression of Col-II was examined through Col-II immunohistochemical staining. Five regions were randomly selected from the slices and the integrated option density values of the Col-II positive area were analyzed by software Image-J (National Institute of Mental Health, Bethesda, Maryland, USA).

### Elisa

Proteins were extracted from the NP tissue of T12-L1 and L1–2 segment at each time point. The concentration of lactic acid (Sigma-aldrich, St. Louis, Missouri USA), aggrecan (BlueGene Biotech, Shanghai, China) and NGF (MyBioSource, San Diego, California, USA) was examined by an ELISA kit according to the manufacturer’s instructions.

### Western blot

Proteins were extracted from the NP tissue of T12-L1 and L1–2 segment at each time point and were quantitated using a protein assay kit (Bio-Rad, Mississauga, Ontario, Canada). Protein samples (30 μg) were fractionated by SDS-PAGE and transferred to nitrocellulose membranes. Immunoblotting was carried out as described [19] by using primary antibodies against Col-I, Col-II (Abcam, Boston, MA, USA) and β-actin (Bioworld Technology, St. Louis Park, MN, USA). For standard Western blotting detection, blots were incubated with HRP-conjugated antibody. Bands were visualized using ECL chemiluminescence (Pierce, Rockford, IL, USA) and quantitated by Scion Image Beta 4.02 (Scion Corporation, NIH).

### qRT-PCR

The tissue samples from the NP tissue of T12-L1 and L1–2 segment at each time point were put into a mortar and grinded with liquid nitrogen. The total RNA was extracted according to the steps on the Qiagen RNA extraction kit instructions. Real time RT-PCR were performed as described previously [19]. The primer sequences of NGF, matrix metallopeptidase 3 (MMP3), matrix metallopeptidase 13 (MMP13), aggrecan and Col-II used for the real-time PCR are presented in Table 1.

**Table 1:** Primers for qRT-PCR

Name	S/AS	Sequence	Tm(°C)	bp
Aggrecan	S	TCCAATGACTCTGGGATCTATC	56	221
	AS	TGGAAGCCGTCCTCATAGGCGG		
NGF	S	GGCCCAATAACGGCTTTTCC	59	182
	AS	TTTAGTCCAGTGGGCTTGGG		
MMP3	S	GGCCGGGGATTTATGGAGAA	60	198
	AS	CTTGAGAAAGGCGGAACCGA		
MMP13	S	ACATCACTCCGGACCGACTA	60	194
	AS	GCTGCAGTGGATCAGCTCTT		
β-actin	S	GAGAAGGCCGGGGCTCACTTGAAG	56	227
	AS	CCGTGGTCATGAGTCCCTCCAC		

*S* sense, *AS* antisense

### Statistical analysis

Data from image analysis were presented as mean ± SEM (structural equation modeling). Statistical comparisons were made using single factor analysis of variance and t test, with *P* < 0.05 being considered significant, *p < 0.05; **p < 0.01; ***p < 0.001.

## Results

Operations were successful in all 18 animals and they were able to walk independently after waking. The wound healing was good in 17 animals; however, a purulent infection in the deep part of the wound was found in one animal in the AL group 1 week after surgery. Therefore, one animal was added to the AL group.

### MRI study

**Fig. 1:**
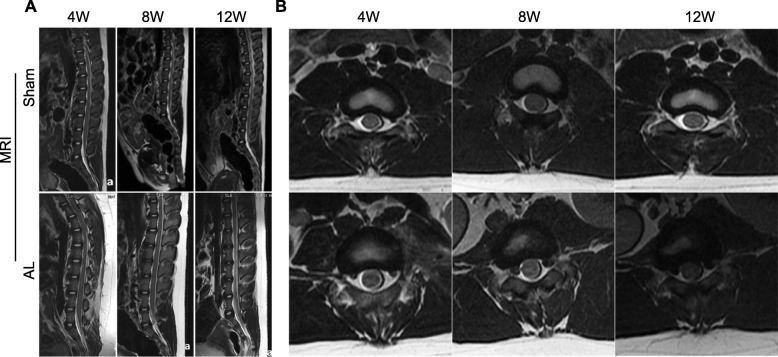
MRI scanning showed obvious intervertebral disc degeneration in annular lesion (AL) surgery group. **a** T2-weighted sagittal MRI scans, showed changes in nuclear volume and signal intensity in disc(T12–L4) 4/8/12 weeks after AL surgery compared with the sham group discs. **b** Axial MRI scans of disc(L1–2) in sham and AL group

There were no obvious degenerations in the sham group (Grade I and II). The discs had a bright, hyperintense white signal intensity in the sagittal T2-weighted images; and the structure of discs was homogeneous with a normal disc height. In the AL group, the grade was significantly higher than those in the sham group as early as 4 weeks postoperative and became more aggressive by 8 and 12 weeks (Grade IV and V). The discs had a hypointense dark black signal intensity; and the structure was inhomogeneous with a moderately decreased disc height (Fig. 1a-b). The change of disc degeneration grade is listed in Table 2.

**Table 2:** The Pfirrmann classification of intervertebral discs in two groups

Grade	4 weeks		8 weeks		12 weeks	
	sham	AL	sham	AL	sham	AL
I	14	0	8	0	3	0
II	22	16	16	4	9	0
III	0	20	0	13	0	2
IV	0	0	0	6	0	7
V	0	0	0	1	0	3

*sham* sham group, *AL* annular lesion group

### General observation and Histopathological analysis

**Fig. 2:**
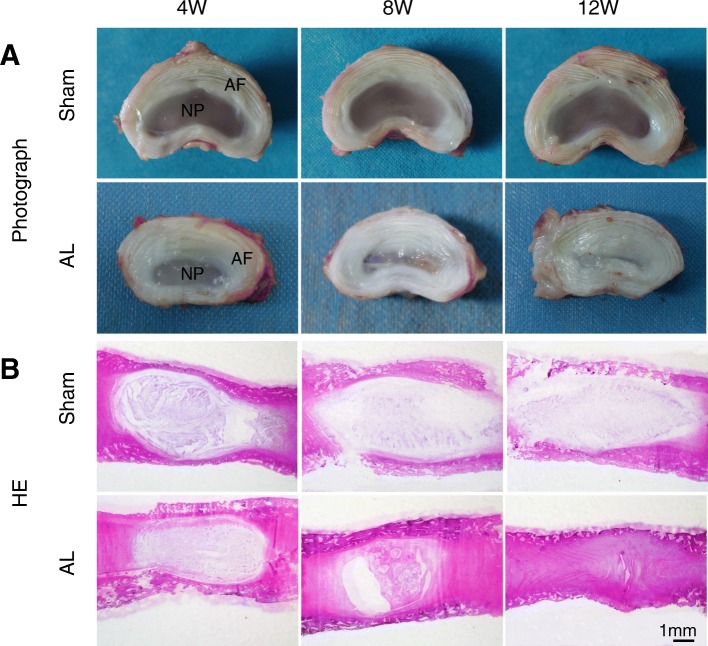
Severe change of the nucleus pulposus (NP) and annulus fibrosus (AF) tissue was found in AL group. **a** Transverse sections through a L1–2 intervertebral disc of sham and AL group 4/8/12 weeks after surgery, showed the obvious degeneration of the NP and AF. **b** HE staining showed the gradually fibrosis of nucleus pulposus in disc of AL group

By coincidence with the MRI results, obvious degeneration of the IVD was found in isolated discs of AL group; and, the height of the IVD showed a progressive decrease after the annular lesion surgery. The boundary between the AF and the NP was gradually unclear, and the gel-like substance was continuously lost and replaced by fibrous tissue. The elasticity and hydrophilicity of the IVD was also reduced. No disc degeneration was found at any of the time intervals in the sham group (Fig. 2a).

**Fig. 3:**
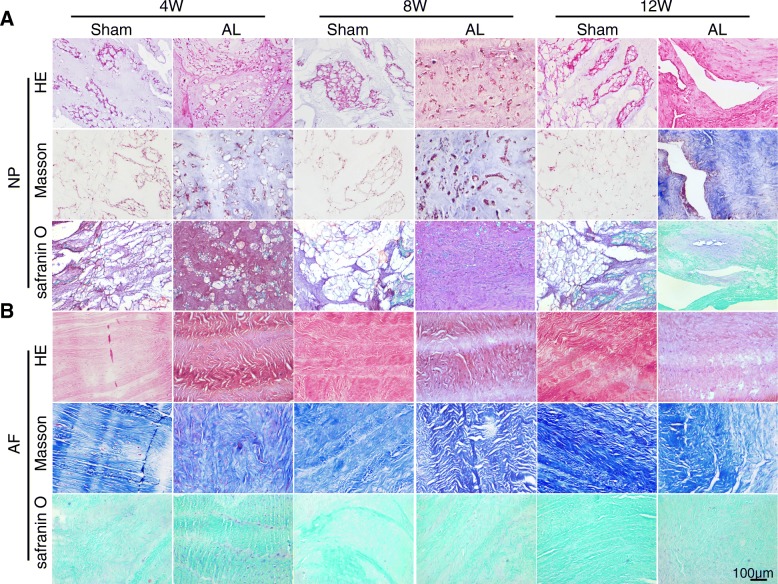
Degeneration of the nucleus pulposus (NP) and annulus fibrosus (AF) tissue cells was found in AL group. **a** HE and Masson and Safarnin O and fast green staining showed severe damage of the NP of L2–3 intervertebral disc at 4, 8 and 12 weeks after lesion, NP tissue was replaced by hyperplastic connective tissue. **b** AF was rupture and disorder in annular lesion group compared with sham group

In the sham group, HE staining showed that AF and NP had no obvious degeneration. However, NP cells gradually decreased, even totally disappeared at 12 weeks postoperatively in the AL group (Fig. 2b). The sham group contained more large vacuoles cells and small cartilage like cells in the nucleus pulposus, however, the number of vacuoles cells reduced and cartilage like cells gradually increased in the AL group (Fig. 3a). Nucleus shrinkage and fibrous tissue hyperplasia occurred at 8 weeks and the degeneration of cartilage cells was also visible. Twelve weeks after postoperatively, the nucleus regions were almost entirely replaced by fibrous tissue and only cartilage cells and fibroblast like cells were found in the NP area (Fig. 3a). Masson staining, Safranin O and fast green staining showed that the fiber ring was arranged in concentric circles with a clear lamellar structure, and the collagen was arranged in grid structure rules in sham group. In the AL group, the boundary of the annulus and nucleus pulposus junction was unclear, the fiber ring was arranged in disorder. The fiber ring was further tortuous, even fractured, and no obvious lamellar arrangement of concentric circle structure was found at 8 and 12 weeks after surgery. The number of fusiform fibroblasts reduced and the number of large cartilage like cells increased in AF (Fig. 3b).

**Fig. 4:**
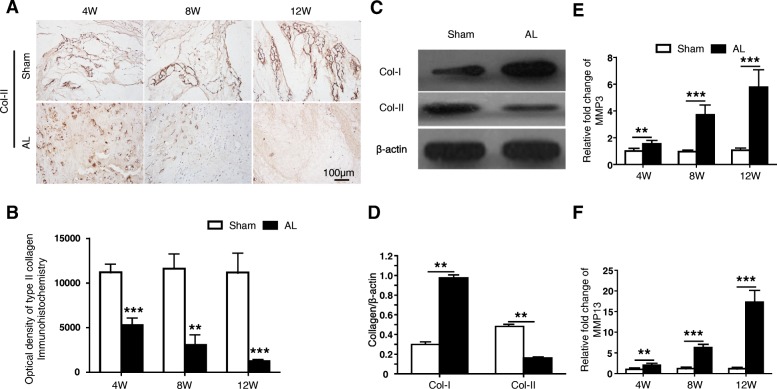
Expression of type II collagen (Col-II) significantly decreased in NP tissue of AL group. **a** Immunohistochemical staining showed the positive area of Col-II in the NP tissue decreased with the degree of degeneration. **b** Statistical analysis of the average optical density of type II collagen immunohistochemical staining, ** *p* < 0.01; *** *p* < 0.001 compared with the sham group at same time point. **c** Western blot showed that the expression of type I collagen (Col-I) was increased, and Col-II decreased in NP tissue of degeneration group 12 weeks after surgery. **d** Statistical analysis of the western blot bands, ** *p* < 0.01 compared with the sham group at same time point. **e**-**f** The gene expression of matrix metallopeptidase 3 and 13 (MMP3 and MMP13) gradually increased over time in AL group compared with sham group at 4/8/12 weeks after surgery

We further examined the expression of Col-II in the NP tissue through Col-II immunohistochemical staining. The notochord and cartilage cells of the NP were positive stained in sham group. However, the Col-II positive staining cells gradually faded with the aggravation of IDD in the AL group; and, almost no positive staining was found at 12 weeks (Fig. 4a-b). The data demonstrated that the Col-II synthesis ability of nucleus pulposus cells significantly decreased in the AL group compared with the sham group.

### Biochemical and molecular biology analysis

Western Blot was used to evaluate the expression of Col-I and Col-II in the central NP tissue at 12 weeks postoperatively. The results showed Col-II was mainly expressed and little expression of Col-I was found in the sham group. While the expression level of Col-II significantly decreased, the expression level of Col-I extremely increased in the AL group (Fig. 4c-d). To investigate the role of MMP3 and MMP13 in extracellular matrix (ECM) degradation of our animal model, we further examined the gene expression of MMP3 and MMP13 in NP tissue in the Sham and AL groups at different time intervals. We found both MMP3 and MMP13 gradually increased in AL group compared with sham group (Fig. 4e-f).

**Fig. 5:**
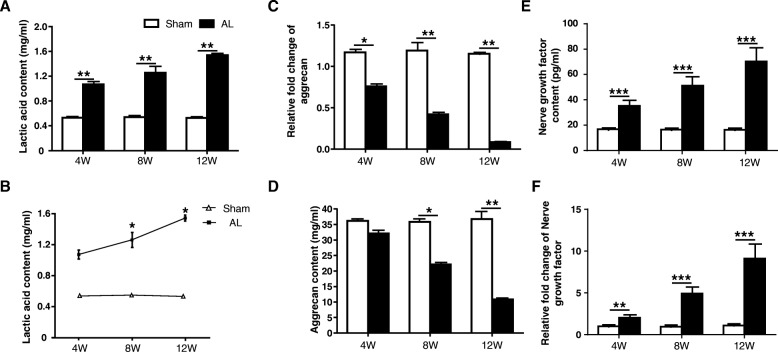
Increased lactic acid and nerve growth factor (NGF) content and extracellular matrix depletion of the intervertebral disc was found in annular lesion (AL) group. **a-b** The content of lactic acid in the nucleus pulposus of L1–2 intervertebral disc was dramatically increased over time in AL group compared with sham group at 4/8/12 weeks after surgery. **c-d** The gene expression and content of aggrecan in the nucleus pulposus of L1–2 intervertebral disc was decreased over time in AL group compared with sham group at 4/8/12 weeks after surgery. **e-f** The gene expression and content of aggrecan in the nucleus pulposus of L1–2 intervertebral disc was decreased over time in AL group compared with sham group at 4/8/12 weeks after surgery. ** *p* < 0.01; *** *p* < 0.001 compared with the sham group at same time point

The changes of lactic acid, aggrecan and NGF in the NP tissue were evaluated by ELISA. The content of lactic acid was significantly higher in the AL group compared with the sham group at each time point. As the degree of disc degeneration increased, the content of lactic acid also gradually rose (Fig. 5a-b). The content of aggrecan gradually decreased in the AL group which was consistent with the changes of the aggrecan gene expression level detected by the qRT-PCR (Fig. 5c-d). The content of NGF gradually increased in AL group compared to the sham group, which was consistent with the NGF qRT-PCR result (Fig. 5e-f).

## Discussion

The IVD is the largest avascular organ in the human body. Nutrients, oxygen and metabolites are diffused through the cartilaginous endplates and the outer annulus with the endplate pathway as the major route [20]. With the aging and/or other factors, insufficient nutrient supply may play an important role in the degeneration of the IVD. When endplate calcification occurs, diffusion between the NP and blood vessels is compromised. With the fall of oxygen levels from decreased diffusion, anaerobic respiration takes place and the pH value within the disc drops as the lactic acid concentration increases, especially in the mid-layer of the disc [7, 20] . The dense matrix affects the outgoing lactic acid which leads to an increase in the acidity of the matrix and surrounding cells. Acid hydrolysis of the annulus creates fissures, leaking lactic acid into surrounding tissue. Persistent acid burn leads to chronic inflammation and discogenic pain [5]. Nachemson [21] reported a significant correlation between preoperative low back pain and low pH measured by a pH-electrode in lumbar rhizopathy surgery. In addition, NGF and its receptor tropomyosin receptor kinase A receptor (TrkA) also has been found to play a critical role in chronic pain associated with IDD [12, 13]. In previous studies, Urban et al. showed that a low pH value of IVD caused a series of damage to the IVD [7]. Increasingly in vitro studies have confirmed that acidic pH conditions mimicking a degenerative IVD may induce a catabolic mechano-response in human NP cells [22]; and may also impair the survival and biological behavior of mesenchymal stem cells (MSC) which may affect the efficacy of MSC-based IVD regeneration [23]. Many factors can cause a low pH environment in an IVD [8, 24]. Lactic acid is the most important factor affecting pH value. The change of lactic acid content in an IVD is closely related to the physiological function of an IVD [7, 8, 25]. Wu et al. [26] concluded that high lactate concentration was a pathogenic factor for disc degeneration in rat nucleus pulposus cells, and lactate metabolism may be a new therapeutic target for disc degeneration. However, no study has reported a lactic acid level in a degenerative disc in vivo, and how lactic acid accumulation impacts disc composition.

Pervious study [5] measured the pH value of IVD by a calibrated micro pH electrode. pH is strongly dependent on lactic acid, however, no study measured the lactic acid in the NP of IVD. Bartels et al. [27] measured the lactate concentrations in anulus. In this study, we used ELISA to measure the concentration of lactic acid of NP. We believe this is the most accuracy way for evaluate the change of lactic acid. The most important extracellular matrix of NP cells are proteoglycan and type II collagen, which is main components of the ECM which maintains the hydrophilicity and physiological function of the IVD. The loss of proteoglycan results in a decrease in the gelatinous substance of the nucleus pulposus, resulting in gradual fibrosis and decreased elasticity [28]. In our study, we directly observed anatomical structure changes of IVD. And we used MRI to evaluate changes in the NP signal, which is closely related to the proteoglycan and type II collagen. Therefore, because the results of imaging, histology and molecular biology were compatible. Our study is more accurate and reliable in evaluating the changes of ECM in degenerated IVD as well as corresponding lactic acid concentration. Overexpression of ECM remodelers, such as MMPs are associated with IDD [29]. The most frequently studied MMPs in disc aging and degeneration are MMP1, MMP3 and MMP13, which degrade different types of collagens [30–33]. Along with MMP3, MMP13 is one of the main proteases described in IDD [34, 35]. In our study, we found both MMP3 and MMP13 gradually increased in AL group compared with sham group, and MMP13 dramatically increased after 12 weeks, which suggests MMP13 may play an important role in matrix components changes of the NP tissue in AL group. Surgical injury to the IVD is a widely used method of inducing disc degeneration. In this study, we used a superficial fibrous ring injury model to induce IDD that is currently recognized as an appropriate method to simulate the natural degeneration of an IVD [36].

## Conclusion

Our study is the first to indicate that increased lactic acid content and ECM depletion occur simultaneously during degeneration of the IVD. Therefore, shunting the intradiscal excessive accumulation of lactic acid out of the IVD may improve the acidic environment and repair disc degeneration [37]. We believe these findings is useful for further research to reduce the content of lactic acid inside of degenerative disc, which can become a novel treatment for degenerative disc disease.

## Data Availability

The datasets supporting the conclusions of this article are included within the article and raw data will be made available from the authors upon reasonable request.

## Abbreviations

AF: Annulus fibrosus
AL: Annular lesion surgery
Col-I: Type I collagen
Col-II: Type II collagen
ECM: Extracellular matrix
HE: Hematoxylin and eosin
IDD: Intervertebral disc degeneration
IVD: Intervertebral disc
MMP13: Matrix Metallopeptidase 13
MMP3: Matrix Metallopeptidase 3
MRI: Magnetic Resonance Imaging
MSC: Mesenchymal Stem Cells
NGF: Nerve growth factor
NP: Nucleus pulposus
qRT-PCR: Quantitative reverse transcription polymerase chain reaction
SEM: Structural Equation Modeling
